# Supplementary material for: Teacher attitudes toward inclusion of students with disabilities in Jeddah elementary schools
Source: PLoS One. 2023 Jan 19;18(1):e0279068. doi: 10.1371/journal.pone.0279068 (PMC9851549; doi:10.1371/journal.pone.0279068)
Supplement: Attachment — Submitted filename: Teacher attitudes PLosOne.docx [file pone.0279068.s003.docx]

**Teacher attitudes toward the inclusion of students with disabilities in Jeddah elementary schools**

Page 18. Line 225

Table 2 shows that all items included in the survey are significantly correlated with the related subscale

The Correlation table always predicts about two factors/items e.g., the weight and height of the body or study hours and GPAs. The way the authors have shown the table is WRONG.

No 1 item has a correlation with other items??

page 18 Line 233 -234

Analysis of variance (ANOVA) was conducted as an inferential statistical method to examine the association between teachers’ demographic characteristics and their perceptions of SEN.

Line 240-241

Further, 43.9% worked at ordinary schools providing private education classes and 40.4% worked in ordinary schools that did not provide private education classes. Only 15.7% worked in integrated schools.

*Comment: if 15% of teachers have been working in special schools, then results do not show true population perceptions authors mentioned Nearly* 67% did not have any contact with students with SEN outside school.

Line 265

A one-way ANOVA between subjects was conducted to examine the effect of the type of 264 school provision (full or partial inclusion, or no provision for inclusion) on teachers’ attitudes toward inclusion. The effect of the independent variable on the dependent one was not found statistically significant at the p < 0.05 level.

*Comment: ANOVA measures the mean differences of three or more groups. The given table does not reflect the ANOVA values. The effect is measured by Regression analysis. Absolutely wrong*

Line 6

Table 6: One-Way analysis of variance (ANOVA) to examine the influence of school types on teachers' attitudes towards the inclusion of students with SEN.

Comment: The table must show the F value, df, P value, and Postdoc if there was a difference in means between the three groups (schools). The given Table 6 is wrong

Table 7 shows Ranking, usually, it is used when data is not normally distributed and a non-parametric test is applied. SPSS itself gives ranking

Overall, inappropriate methodology, ( they should use multistage cluster sampling ) lack of content validity of study tool, and wrong statistical analysis make this study to be rejected

A good option is to use the qualitative method.
