# Supplementary material for: Teacher attitudes toward inclusion of students with disabilities in Jeddah elementary schools
Source: PLoS One. 2023 Jan 19;18(1):e0279068. doi: 10.1371/journal.pone.0279068 (PMC9851549; doi:10.1371/journal.pone.0279068)
Supplement: Attachment — Submitted filename: Responses to Reviewers.docx [file pone.0279068.s004.docx]

**Comments to the Author**
• Language should be reviewed in all parts of the article, many paragraphs were not structured in a research based format an example of this (the second paragraph on page 4 under “Importance and Effectiveness of Using Assistive Technology”) the whole paragraph is lacking good structure, proper connectors, and most important the lack of full sentences. Done. Point ADDRESSED: The entire article was revised and sent to the Writing Center Service of SAGE OPEN, as shown.
• The use of “Qualitative data” in many places is not appropriate even if the researcher wanted to express “Quality data”

Done. Point ADDRESSED: This research ensured that the reference to quantitative data was stated appropriately, and reference to qualitative data was omitted.
• The main following sections  “Main aim of this study, Research questions, Research design, sampling, data collection and procedures”  are all presenting the same idea with high redundancy among them, either join under main headings or rewrite to define in a better way

Done. Point ADDRESSED: The researcher reorganized the headings appropriately.
• The teachers’ socio-demographic profile can be easily presented by a detailed table that includes all numbers without all the description and redundancy by repeating all numbers again.

Done. Point Addressed: A detailed table is provided, including all numbers without redundancy.
• This section “socio-demographic profile “ should been moved under sampling to describe the sample especially that it is not going to be discussed under any of the research questions.

Done. Point Addressed: The socio-demographic profile has been moved under the sampling and data analysis sections.
• Almethen (2017) which is the tool that has been adapted and used in this research, has the following:
o The tool is not referenced in the list of references

Done. Point Addressed: This has been included in the list of reference.
o The tool is not introduced in anyway, the content, number of items, factors or sections if applicable, validity and reliability of the tool (just citing the basic types addressed)

Done. Point Addressed: The tool is now introduced with discussion of its validity and reliability.
o I think that the Almethen (2017) has been applied I Saudi Arabia, accordingly how is this research different from that one especially that no comparisons have been made later on with it in the discussion part.

Done. Point Addressed: The results of this study were compared with those of studies conducted in Saudi Arabia.
• After a quick review of current publications I found the following:
o Alharbi, S. A. (2018). Special education teachers' knowledge and use of assistive technology for inclusive classrooms in Saudi Arabia (Doctoral dissertation, Saint Louis University).
o Alanazi, A. S. (2019). General and Special Education Teachers’ Attitudes Toward Using Assistive Technology in Classrooms for Students with Autism Spectrum Disorder in Saudi Arabia (Doctoral dissertation, Concordia University Chicago).
o Alasmari, A. A. (2020). Special Education Teachers’ Knowledge and Use of Assistive Technology with Students with Expressive Language Disorders in Saudi Arabia (Doctoral dissertation, Saint Louis University).
o Alanazi, A. (2020). Special Education Teachers’ Knowledge of Using Assistive Technology with Students with Autism Spectrum Disorder. Technium: Romanian Journal of Applied Sciences and Technology, 2(7), 54-63.
o Alsawalem, I. M. N. (2019). TEACHERS’ATTITUDES TOWARDS USE OF INFORMATION COMMUNICATION TECHNOLOGY WITH STUDENTS WITH INTELLECTUAL DISABILITY IN SAUDI ARABIAN SCHOOLS (Doctoral dissertation, University of Newcastle).
• None of the previous published and up-to-date research has been cited in this research article although that all is highly connected to the current topic.

Done. Point Addressed: All the aforementioned studies are now cited in this study.
• Reformatting of Tables, especially table 8

Done. Point Addressed: New formatting has been applied to many tables, including Table 8.
• The researcher did not mention anything related to the calculation of scale used (upper and lower values) how percentages or means will be used, cut-off points and how did they specify them.

Done. Point Addressed: Details of the calculation of scale used in this study have been provided.
• In page 21 the researcher presented that the sample averaged 3.05, can we really get to this generalization from only one item, were there any other items related to the environment or not?

Done. Point Addressed: This was modified in the revision of this paper.
• All results related to the first question should be re-examined and the generalizations perceived by the researchers should be explained and supported  in a better way especially with the lack of information related to the tool itself. Furthermore, it is impossible to accept such results with minimal background information about the tool and it is components.
• One simple questions, if the sample presented such a high level of skills and knowledge what is the need for further training, especially that 90% of them are requesting it, such a contradiction must be rationalized here.

Done. Point Addressed: The questions were re-examined and the results have been changed.
•  The term “competencies” should be defined, especially that competencies has been fully substituted by skills and knowledge.

Done. Point Addressed: This term is now defined.
• Discussion should be reviewed  according to previous changes in order to be in understood and comprehended.
Done. Point Addressed: The discussion has been changed accordingly.

Reviewer: 2
Comments to the Author
Overall, well written and easy to follow.
There are statements like, "In contrast, improvements in special education in the USA got underway when legislators mandated that AT services and devices would serve as important cornerstones in all learners’ Individualized Educational Plan (IEP)" which are simply not true. AT is NOT mandated in the USA. If a student needs AT it is provided for them but it is not a requirement.

Done. Point Addressed: This has been modified as indicated.
Also, I am not sure what you what to study..."special education teachers' perceptions about their AT competencies; their
interest in receiving AT training OR special education teachers' usage of AT. These are two different things.

Done. Point Addressed: The research mainly aimed to evaluate their competency in using AT and their preference for receiving training, if needed.
The second research question is not open ended.

Done. Point Addressed: That is correct.
If you "adapted the instrument" it becomes invalid and unreliable. Thus your results are not valid. What type of scale did the survey use?

Done. Point Addressed: This has been addressed as stated in the high reliability table.
I do not see how a recommendation and conclusion about Autism can be made because the study was not about a specific disability.

Done. Point Addressed: The collected data were reanalyzed, and the results have been modified accordingly.
Simply training special ed teachers in the use of AT will not necessarily improve student with exceptionalities' academic progress. It may work for some but not others. I do not feel that your study proved this.

Done. Point Addressed: If teachers are prepared and trained in effectively using AT, they will positively reflect on their students with disabilities. The major problem we face today is that some teachers do not know how to use basic devices to assist their students.
On p. 10 you state that you are suing a quantitative methodology but on p.11 you state you received "qualitative data" which is correct?

Done. Point Addressed: This has been modified.
p. 12 I am unclear how the "....perceptions of professional development..." aligns to your research questions. The entire survey should align to the research questions. From what is stated here it appears that only section four does this.

Done. Point Addressed: There is a separate section specified for professional development, which is now clearly stated in the analysis and discussion sections.
I did not see any theory discussed or aligned to the research questions.

Done. Point Addressed: The theory has been added.
Reference page is not in correct 7th APA format

Done. Point Addressed: 7^th^ APA format has been applied to the references.

Reviewer: 3

Comments to the Author
Overall, this is an interesting paper that can make a contribution to the research on assertive technology and special education. This article reports the findings of a quantitative study conducted with 64 special education teachers working in schools located in a specific area on Saudi Arabia. This is an interesting topic and the paper as a whole would be of interest to the journal audience. However, I would recommend the author(s) to revise the article and do some revisions before it can reach the standards to be published in SAGE Open.

Done. Point Addressed: This paper has been revised, and I hope has reached the standard of publication required by SAGE Open.
The author(s) clearly summarize the aim, methods and introduce briefly some relevant findings of the article in the abstract. This is followed by the introduction which presents an account of the literature that presents the field of assertive technology and its implementation in the field of special education, putting special attention to the disparities in different countries such as the United States of America, United Kingdom or Saudi Arabia itself. Special attention is paid in this section to the importance of assertive technology on education and the teachers’ training opportunities in this regard. Whereas the literature is pertinent and relevant, it does not report strong evidence to frame the present study since it presents slight affirmations from previous research. In this regard, the manuscript would benefit from offering the particular contributions of the previous literature; this would allow to strengthen the arguments and to highlight what the gap is. It also would benefit to be updated.

Done. Point Addressed: The researcher has cited these five new articles and dissertations and used them in the discussion.

Moreover, the coherence on the spelling should be considered, since Assertive Technology is written both with and without capital letters all over the text.

Done. Point Addressed: This spelling has been made consistent throughout.

Furthermore, “KSA” appears for the first time on page 8, but it should be explained before.

Done. Point Addressed: This has been modified throughout the article.
The Methodology section can also be strengthened by clarifying some key points. The research design can be justifying (why does it fit the purpose of the study?).

Done. Point Addressed: The section on the research design explains why the researcher chose this design.

The sampling section is scarce; maybe the author(s) could give and insight of the participants through a brief table that summarizes key information (gender, age, expertise, and all the information that the figures 1 to 5 and tables 1 and 2 collect).

Done. Point Addressed: This information is now stated in Table 1.

Moreover, the procedure section, could be rewritten for better understanding of what was the exact paths the researcher(s) made in this sense.

Done. Point Addressed: the discussion of the procedure has been clarified to indicate that the respondents are adults, and we sent them a link to the survey via their WhatsApp or email address.

Some information in this section could be better explained: why 6 of the surveys were deleted? Did the participants withdraw? More clear explanation will benefit the soundness and the transparency of the manuscript.

Done. Point Addressed: Because they did not complete their answers, their surveys were considered missing data. In addition, we encountered two major problems: First, participants did not always complete their questionnaires when they clicked and opened the survey, and some participants appear to have answered without reading the questions thoroughly, which created discrepancies in the data analysis section.

Another issue that should be clarified refers to the ethics, was the research design approved by an ethic committee?

Done. Point Addressed: The letter of the ethics committee has been attached.
Whereas in the abstract and in other sections the paper refers to autism, it is not clear why the study ends focusing in this particular disability, since it arises on page 16 for the first time.

If the target autism, the introduction should include this information. This should be more clearly explained, as well as the criteria for choosing in that particular disorder.

Done. Point ADDRESSED: The results were changed according to the data analysis.

Moreover, information regarding the data analysis is scarce, it is not clear how was the analysis strategy.

Done. Point ADDRESSED: The researcher revised the data analysis, and the results have been included accordingly.
The information presented in the results section seems a bit confusing. The information of section two and section three in the “data analysis” part could be better present as results if the author(s) organize the information accordingly. Indeed, if the demographic information of the participants was not an objective of the research (now in “section one” within “data analysis” par), it seems confusing to present it along with what this reviewer understands that are the main results of the research.

Done. Point ADDRESSED: The researcher has reorganized them in a better way to help readers easily follow up the data analysis and discussion.
Discussion reflects on the literature and the claims in this section are supported by the results, although there is no reference to the limitations of the study, which should be also acknowledged.

Done. Point ADDRESSED: This has been added to the paper.
Hope these comments may be useful for the author(s).

These comments helped me understand my paper much better. I greatly appreciate your effort and generosity in sharing your knowledge and accumulated experience with me.

Thank you very much again.
